# C1GALT1 predicts poor prognosis and is a potential therapeutic target in head and neck cancer

**DOI:** 10.1038/s41388-018-0375-0

**Published:** 2018-06-21

**Authors:** Mei-Chun Lin, Pin-Hui Chien, Hsin-Yi Wu, Syue-Ting Chen, Hsueh-Fen Juan, Pei-Jen Lou, Min-Chuan Huang

**Affiliations:** 1 Department of Otolaryngology, National Taiwan University Hospital, Hsin-Chu Branch, Hsinchu, Taiwan; 2 National Taiwan University Cancer Center, Taipei, Taiwan; 3 Instrumentation Center, National Taiwan University, Taipei, Taiwan; 4 Graduate Institute of Anatomy and Cell Biology, College of Medicine, National Taiwan University, Taipei, Taiwan; 5 Department of Life Science, Graduate Institute of Biomedical Electronics and Bioinformatics, National Taiwan University, Taipei, Taiwan; 6 Department of Otolaryngology, National Taiwan University Hospital and College of Medicine, Taipei, Taiwan

## Abstract

Core 1 β1,3-galactosyltransferase (C1GALT1) controls the crucial step of GalNAc-type O-glycosylation and is overexpressed in various human malignancies. However, its role in head and neck squamous cell carcinoma (HNSCC) remains unclear. Here we demonstrate that C1GALT1 expression is upregulated in HNSCC tumors and is associated with adverse clinicopathologic features. Moreover, high C1GALT1 expression predicts poor disease-free and overall survivals. C1GALT1 overexpression enhances HNSCC cell viability, migration, and invasion, which can be reversed by erlotinib. Silencing of C1GALT1 suppresses the malignant behavior both in vitro and in vivo. Mass spectrometry and lectin pull-down assays demonstrate that C1GALT1 modifies O-glycans on EGFR. Blocking O-glycan elongation on EGFR by C1GALT1 knockdown decreases EGF-EGFR binding affinity and inhibits EGFR signaling, thereby suppressing malignant phenotypes. Using molecular docking simulations, we identify itraconazole as a C1GALT1 inhibitor that directly binds C1GALT1 and promotes its proteasomal degradation, leading to significant blockade of C1GALT1-mediated effects in HNSCC cells in vitro and in vivo. Collectively, our findings demonstrate a critical role of O-glycosylation in HNSCC progression and highlight the therapeutic potential of targeting C1GALT1 in HNSCC treatment.

## Introduction

Head and neck squamous carcinoma (HNSCC) consists of squamous carcinoma arising in the oral cavity, oropharynx, hypopharynx, and larynx. It is the fourth leading cancer among Taiwanese men and accounts for >600,000 cases annually worldwide [1]. The main state of treatment for locally advanced HNSCC is surgical resection followed by chemoradiotherapy. However, the 5-year survival rate remains below 50% despite multidisciplinary treatments [2]. Timeless efforts to unravel the pathogenesis of HNSCC has been made but the progress in targeted or personalized therapy is limited [3, 4].

Glycosylation is one of the most common post-translational modification in mammalian cells and is critical in regulating physiological processes, including cell adhesion, migration, cell–cell recognition, and immune surveillance [5]. Glycans in normal cells are constructed in an orderly manner involving substrate-specific glycosyltransferases [6]. Altered glycosylation during malignant transformation was first discovered 60 decades ago and later recognized as a hallmark in human cancers [7]. GalNAc-type O-glycosylation is the most common type of O-glycosylation and is initiated by the transfer of *N*-acetylgalactosamine (GalNAc) to a serine or threonine residue, forming the Thomsen-nouvelle (Tn) antigen [8]. This reaction is catalyzed by a family of polypeptide GalNAc transferases (GALNTs), consisting of 20 members in humans [9]. Following the initial step, C1GALT1 is the only enzyme that transfers UDP-galactose to Tn antigen to form core 1 structure, which is also called Thomsen-Friedenreich (T) antigen. T antigen is a precursor for many extended GalNAc-type O-glycans on cell surfaces and secreted glycoproteins [10]. De novo appearance of short O-glycans, such as Tn, sialyl-Tn, and T antigens, features aberrant glycosylation in malignant tumors [11], including HNSCCs [12]. Although mechanisms that cause the generation of tumor-associated glycans are not yet fully understood, expression and localization of glycosyltransferases undoubtedly play an important role [13].

*C1galt1* knockout is embryonically lethal in mice, which exhibit severe thrombocytopenia and bleeding tendencies [14]. Defects of C1GALT1-specific chaperone, COSMC, in humans cause Tn syndrome, which is manifested by erythrocyte polyagglutination [15]. We previously found that C1GALT1 is overexpressed in hepatocellular carcinoma (HCC), colorectal cancer, and breast cancer [16–18]. Moreover, C1GALT1 regulates O-glycosylation of MET and FGFR2 in HCC and colorectal cancer cells, respectively. In prostate cancer cells, C1GALT1 regulates EGFR O-glycosylation to enhance galectin-4-mediated phosphorylation of EGFR [19]. Although C1GALT1 controls many cellular behaviors and EGFR serves as a therapeutic target in several malignancies, including HNSCC, lung cancers, and colon cancers, the therapeutic potential of targeting C1GALT1 and its effect on EGFR signaling in HNSCC remain unclear.

In this study, we unravel the expression and function of C1GALT1 in HNSCC. We are the first to provide mass spectrometry (MS)-based evidence showing that EGFR carries GalNAc-type O-glycans which can be modified by C1GALT1. Moreover, silencing of C1GALT1 inhibits the ligand-binding affinity and phosphorylation of EGFR. Importantly, using genetic or small molecule pharmacologic approach, our results suggest that C1GALT1 is an attractive therapeutic target for HNSCC.

## Results

### C1GALT1 is overexpressed in HNSCC tumors and high C1GALT1 expression predicts poor prognosis

**Fig. 1:**
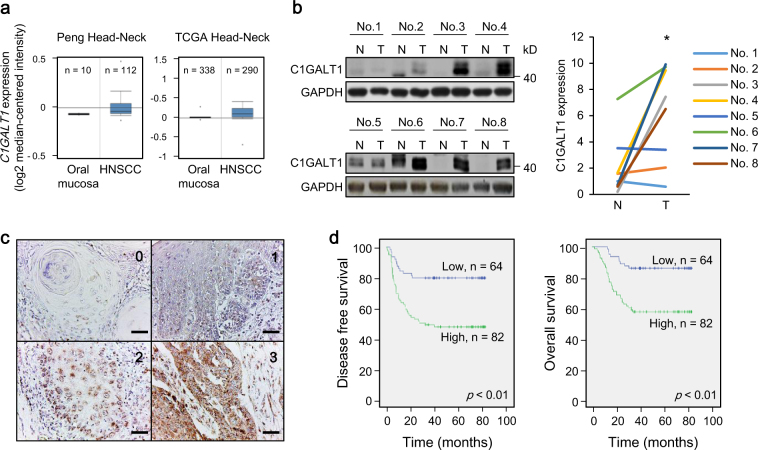
C1GALT1 is overexpressed in HNSCC tumors and high C1GALT1 expression predicts poor prognosis. **a** *C1GALT1* messenger RNA expression in HNSCC. Data are retrieved from Peng Head-Neck and TCGA Head-Neck in the Oncomine database (https://www.oncomine.org). **b** Left panel, western blot analysis of C1GALT1 expression in paired HNSCC tumor tissues (T) with adjacent non-tumor mucosa (N) from 8 patients. GAPDH was an internal control. Right panel, C1GALT1 expression was quantified and analyzed by paired Student’s *t*-test. *P < 0.05. **c** Scores of C1GALT1 expression from 0 to 3 in HNSCC tissues analyzed using immunohistochemistry. Scale bars, 50 µm. **d** Kaplan–Meier survival analysis. Patients with follow-up period over 36 months are included (*n* = 146). Left and right panels indicate disease-free and overall survivals, respectively

To evaluate the expression of C1GALT1 in clinical samples, we first searched public databases (https://www.oncomine.org) and found that C1GALT1 is overexpressed in HNSCC tissues compared with normal oral mucosa (Fig. 1a). To confirm the public complementary DNA microarray data, we performed western blot analysis and found that C1GALT1 is significantly overexpressed in HNSCC tissues compared with adjacent non-tumor parts (*n* = 8, Fig. 1b). We then performed immunohistochemistry of HNSCC tumors (*n* = 153) and scored the staining intensity from 0 to 3 (Fig. 1c). Higher C1GALT1 expression was significantly associated with presence of distant metastasis, lymphovascular invasion, higher nodal stages, and higher histological grades (Table 1). For Kaplan–Meier analysis, we further classified C1GALT1 scores 0–1 and scores 2–3 as low and high expression, respectively. Results showed that high C1GALT1 expression was significantly associated with poor disease-free survival and overall survival (Fig. 1d). Cox regression analysis showed that C1GALT1 was an independent predictor for poor overall survival and disease-free survival (Supplementary Tables 1 and 2). These results suggest that C1GALT1 is overexpressed in HNSCC tumors and is an independent prognostic factor for poor survivals.

**Table 1:** Correlation between C1GALT1 intensity with clinicopathologic characteristics

Clinicopathologic characteristics		No.	C1GALT1 intensity mean (SD)	*P* value
Age	<50	49	1.71 (0.89)	0.92
	≧50	104	1.73 (0.96)	
T	1–2	77	1.58 (0.91)	0.06
	3–4	76	1.87 (0.94)	
N	0–1	112	1.57 (0.92)	**<0.001**
	2–3	41	2.15 (0.85)	
M	0	129	1.61 (0.91)	**<0.001**
	1	24	2.33 (0.82)	
Grade	1	62	1.32 (0.88)	**<0.00001**
	2–3	91	2.00 (0.87)	
LVI	−	112	1.55 (0.94)	**<0.001**
	+	41	2.19 (0.75)	
PNI	−	91	1.64 (0.95)	0.16
	+	62	1.85 (0.90)	

Bold *P* values indicate statistical significance (*P* < 0.05)

*LVI* lymphovascular invasion, *PNI* perineural invasion

### C1GALT1 promotes malignant phenotypes in HNSCC cells

**Fig. 2:**
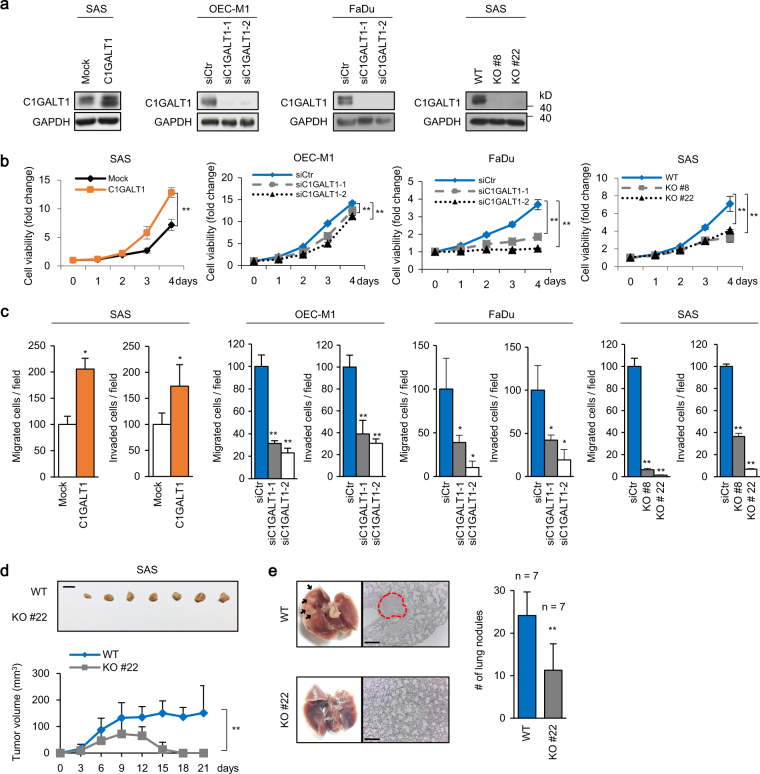
C1GALT1 promotes malignant phenotypes in HNSCC cells. **a** Western blots showing overexpression of C1GALT1 in SAS cells, knockdown of C1GALT1 in OEC-M1 and FaDu cells, and knockout of C1GALT1 in SAS cells. SAS cells were transfected with empty pcDNA3.1 (Mock) or *C1GLAT1*/pcDNA3.1A (C1GALT1). OEC-M1 and FaDu cells were transfected with non-targeting siRNA (siCtr) or two independent siRNAs against *C1GALT1* (siC1GALT1-1 and siC1GALT1-2). These cells were transfected for 48 h before further experiments. C1GALT1 was knocked out in SAS cells with the CRISPR/Cas9 system. C1GALT1 in parental cells (WT) and two C1GALT1 knockout clones (KO #8 and KO #22) were shown. GAPDH was an internal control. **b** Effects of C1GALT1 on viability of HNSCC cells. Cell viability was analyzed using MTT assays at different time points as indicated. Data are analyzed by Student’s *t*-test. **P < 0.01. **c** Effects of C1GALT1 on migration and invasion of HNSCC cells. Transwell migration and Matrigel invasion assays were performed to analyze cell migration and invasion, respectively. Results are represented as mean ± SD. Data are analyzed by Student’s *t*-test. *P < 0.05. **P < 0.01. **d** Effects of C1GALT1 knockout on tumor growth in vivo. Wild-type (WT) or C1GALT1-knockout (KO #22) SAS cells (5 × 10^6^) were injected subcutaneously into NOD-SCID mice. Upper panel, images of tumor xenografts. Scale bar, 1 cm. Lower panel, tumor growth curves. Results are represented as mean ± SD. Data are analyzed by Student’s *t*-test. **P < 0.01. **e** Effects of C1GALT1 knockout on tumor metastasis in vivo. Wild-type (WT) or C1GALT1-knockout (KO #22) SAS cells (1 × 10^6^) were injected into the tail vein of NOD-SCID mice. Left panel, representative images and HE staining of lungs. Arrows indicate metastatic tumor nodules in lungs. Right panel, numbers of tumor nodules in lungs of mice injected with WT or KO #22 SAS cells. Results are represented as mean ± SD. Data are analyzed by Student’s *t*-test. **P < 0.01

To investigate effects of C1GALT1 on HNSCC cells, we analyzed viability, migration, and invasion using C1GALT1 overexpressing, knockdown, or knockout cells. The establishment of these cells was confirmed by western blotting (Fig. 2a). MTT assays showed that C1GALT1 overexpression significantly increased viability of SAS cells (Fig. 2b). By contrast, C1GALT1 knockdown significantly decreased viability of OEC-M1 and FaDu cells. C1GALT1 knockout in SAS cells also significantly decreased viability. Transwell migration and Matrigel invasion assays showed that C1GALT1 overexpression significantly increased while C1GALT1 knockdown and knockout significantly decreased migration and invasion of HNSCC cells (Fig. 2c). To evaluate the effect of C1GALT1 on tumor growth and metastasis, we performed a mouse xenograft model by injecting SAS cells into NOD-SCID mice subcutaneously or through the tail vein, respectively. The results showed that C1GALT1 knockout significantly decreased tumor growth and metastasis (Fig. 2d, e). Echoing with the clinicopathologic data, these results indicate that C1GALT1 promotes malignant behaviors in HNSCC cells.

### C1GALT1 regulates phosphorylation, EGF-binding affinity, and O-glycosylation of EGFR to enhance malignant phenotypes in HNSCC cells

**Fig. 3:**
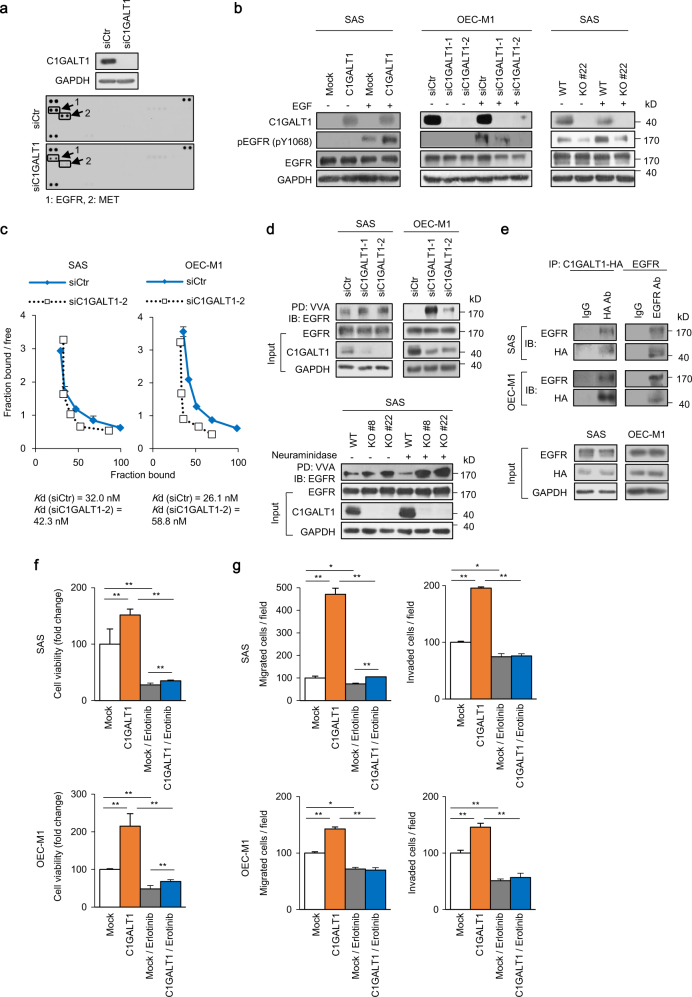
C1GALT1 regulates phosphorylation, EGF-binding affinity, and O-glycosylation of EGFR in HNSCC cells. **a** Effects of C1GALT1 on phospho-RTKs. SAS cells were transfected with non-targeting small interfering RNA (siRNA) (siCtr) or siRNA against *C1GLAT1* (siC1GALT1-2) for 48 h and then treated with 10% FBS for 5 min after 12-h starvation. Upper panel, western blots showing C1GALT1 knockdown in SAS cells. Lower panel, phospho-RTK array. Decreased phospho-EGFR and phospho-MET were indicated. **b** Effects of C1GALT1 on EGF-induced phosphorylation of EGFR. Tyrosine phosphorylation of EGFR (pY1068) was analyzed in C1GALT1 overexpressing SAS cells, C1GALT1 knockdown OEC-M1 cells, and C1GALT1 knockout SAS cells. Cells were starved for 4 h and then treated with (+) or without (−) EGF (10 ng/mL) for 5 min. GAPDH was an internal control. **c** Scatchard plots of ligand-binding assays. SAS and OEC-M1 cells were transfected with non-targeting siRNA (siCtr) or siRNA against *C1GLAT1* (siC1GALT1-2) and collected 72 h later for ligand-binding assays. The *K*d was estimated by the binding data. **d** VVA pull-down assays of EGFR. Upper panel, changes in O-glycans on EGFR in C1GALT1 knockdown SAS and OEC-M1 cells. SAS and OEC-M1 cells were transfected with non-targeting siRNA or siRNAs against *C1GALT1* (siC1GALT1-1 and siC1GALT1-2). Cell lysates were incubated with VVA-conjugated beads for 18 h and immunoblotted with an anti-EGFR antibody. Input EGFR, C1GALT1, and GAPDH were shown. Lower panel, changes in O-glycans on EGFR in C1GALT1 knockout SAS cells. Cell lysates were treated with or without neuraminidase, which was used to remove sialic acids. **e** Co-immunoprecipitation assays of C1GALT1 and EGFR. SAS and OEC-M1 cells were transfected with *C1GALT1*-HA/pcDNA3.1A for 48 h and collected. Lysates were immunoprecipitated (IP) with anti-HA or anti-EGFR antibody, as indicated, and then immunoblotted (IB) with anti-EGFR or anti-HA antibody. **f** SAS and OEC-M1 cells were transfected with empty pcDNA3.1 (Mock) or *C1GLAT1*/pcDNA3.1A (C1GALT1), treated with solvent control or 70 µM erlotinib, and then subjected to MTT assay. Cell viability at day 4 was shown. Data are analyzed by Student’s *t*-test. **P < 0.01. **g** SAS and OEC-M1 cells were transfected with empty pcDNA3.1 (Mock) or *C1GLAT1*/pcDNA3.1A (C1GALT1), treated with solvent control or 70 µM erlotinib, and then subjected to transwell migration, and Matrigel invasion assays. Data are analyzed by Student’s *t*-test. *P < 0.05. **P < 0.01

Previous studies revealed that modification of O-glycans on receptor tyrosine kinases (RTKs) affects their signaling pathways and cancer cell behaviors [16, 17, 20, 21]. To identify the major protein substrates of C1GALT1 that mediate phenotypic changes in HNSCC cells, we first performed phospho-RTK array assays. The result showed that C1GALT1 knockdown or knockout primarily decreased phosphorylation of EGFR and MET (Fig. 3a and Supplementary Fig. 1a). Because EGFR is overexpressed in HNSCC tumors and its signaling pathways play a crucial role in cell survival and invasiveness [22], we focused on the effect of C1GALT1 on EGFR. Our data showed that C1GALT1 overexpression in SAS cells increased EGF-induced phosphorylation of EGFR at Y1068 (Fig. 3b, left panel). By contrast, C1GALT1 knockdown decreased the EGFR signaling in OEC-M1 (Fig. 3b, middle panel) and FaDu cells (Supplementary Fig. 1b, left panel). Moreover, C1GALT1 knockout also decreased the EGFR signaling in SAS cells as shown in two independent clones (Fig. 3b, right panel and Supplementary Fig. 1b, right panel).

To understand the mechanism that mediates changes in EGFR signaling, we examined whether C1GALT1 regulates EGF-binding affinity of EGFR in HNSCC cells using ligand-binding assays. The scatchard plots showed that the dissociation constant (*K*d) of EGF-EGFR in C1GALT1 knockdown and control SAS cells is 42.3 and 32.0 nM, respectively (Fig. 3c). In OEC-M1 cells, the *K*d in C1GALT1 knockdown and control cells is 58.8 and 26.1 nM, respectively.

C1GALT1 transfers galactose to Tn antigen to form T antigen. Decreased C1GALT1 causes accumulation of Tn antigens, which is detected by vicia villosa agglutinin (VVA) lectin (Supplementary Fig. 1c). To detect the effect of C1GALT1 on EGFR O-glycosylation, we performed VVA pull-down assays. The results showed that C1GALT1 knockdown in SAS and OEC-M1 cells (Fig. 3d, upper panel) as well as C1GALT1 knockout in SAS cells (Fig. 3d, lower panel) increased Tn antigens on EGFR, indicating that O-glycans on EGFR were modulated by C1GALT1. Increased Tn antigens on cellular proteins of C1GALT1 knockdown and knockout cells were shown in Supplementary Fig. 1d. To confirm the presence of GalNAc-type O-glycans, EGFR from wild-type and C1GALT1-knockout SAS cells was purified (Supplementary Fig. 1e) and analyzed by LC-MS/MS. The results showed one peptide carrying single HexNAc on EGFR from wild-type SAS cells, and additional four peptides with single HexNAc from C1GALT1 knockout cells (Supplementary Table 3). Consistent with VVA pull-down assay results, these results indicate that EGFR in SAS cells carries elongated GalNAc-type O-glycans, which are modified by C1GALT1. To further support EGFR as protein substrate of C1GALT1, we performed co-immunoprecipitation assays in SAS and OEC-M1 cells and showed that C1GALT1 was associated with EGFR (Fig. 3e).

To evaluate the role of EGFR in C1GALT1-mediated effects, we treated SAS cells with an EGFR tyrosine kinase inhibitor (TKI) erlotinib and then analyzed its effects on cell viability, migration, and invasion. Results showed that erlotinib significantly reversed the C1GALT1-increased viability, migration, and invasion in SAS and OEC-M1 cells (Fig. 3f, g), suggesting a critical role of EGFR in C1GALT1-mediated malignant behaviors. Taken together, these results indicate that C1GALT1 enhances EGF-binding affinity and phosphorylation of EGFR by modifying O-glycosylation of EGFR in HNSCC cells, thereby leading to increased malignant phenotypes.

### Itraconazole binds C1GALT1 to promote its degradation

**Fig. 4:**
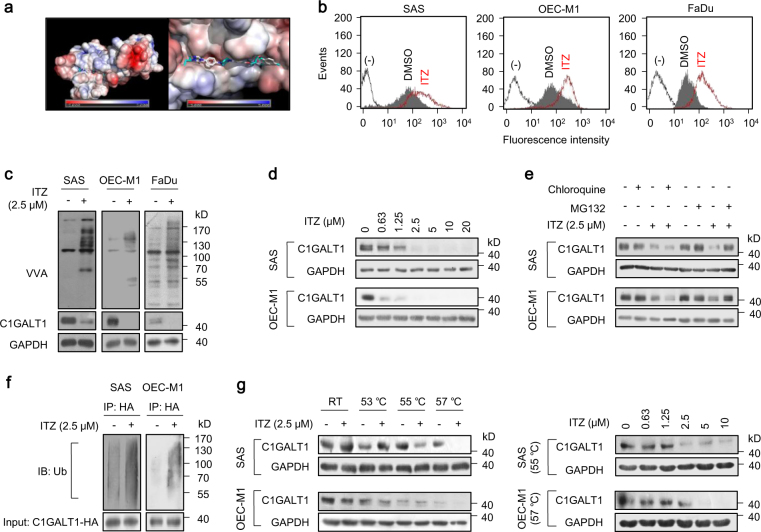
Identification of itraconazole as a C1GALT1 inhibitor. **a** Molecular docking analysis of itraconazole and C1GALT1. Right panel indicates the enlarged binding surface of C1GALT1 and itraconazole. **b** Effects of itraconazole on Tn antigens of the cell surface. Flow cytometry with FITC-VVA on surfaces of SAS, OEC-M1, and FaDu cells treated with solvent control DMSO or 2.5 μM itraconazole (ITZ) for 48 h. **c** Western blot analysis of Tn antigens on cellular proteins and C1GALT1 in SAS, OEC-M1, and FaDu cells treated with DMSO or 2.5 μM itraconazole (ITZ) for 48 h. GAPDH was an internal control. **d** Effects of itraconazole on C1GALT1 protein levels at various concentrations, as indicated. SAS and OEC-M1 cells were treated with DMSO or itraconazole (ITZ) for 48 h and collected for western blot analysis. **e** Degradation pathways of C1GALT1 mediated by itraconazole. Cells were treated with 2.5 μM itraconazole (ITZ) for 24 h, and then incubated with 10 μM chloroquine or 20 μM MG132 for 6 h, as indicated. C1GALT1 levels were analyzed by western blot analysis. **f** Effects of itraconazole on ubiquitination of C1GALT1. SAS and OEC-M1 cells were transfected with *C1GALT1*-HA/DNA3.1A for 48 h and then treated with 2.5 μM itraconazole (ITZ) and 20 μM MG132 for 6 h. Cell lysates were immunoprecipitated (IP) with anti-HA antibody and then immunoblotted (IB) with anti-ubiquitin antibody. C1GALT1-HA in whole cell lysates (input) was shown. **g** Effects of itraconazole on thermal stability of C1GALT1 analyzed using cellular thermal shift assays. Left panel, SAS and OEC-M1 cells were treated with DMSO or 2.5 μM itraconazole (ITZ) for 2.5 h. Cell lysates were incubated at room temperature (RT), 53, 55, or 57 °C for 3 min, followed by cooling at RT for 3 min. Right panel, cells were treated with DMSO or itraconazole (ITZ) at different concentrations, as indicated, for 2.5 h and cell lysates were incubated at 55 or 57 °C. GAPDH was the internal control. (−) unstained cells

To search for C1GALT1 inhibitors, we first modeled the structure of C1GALT1 and then performed molecular docking simulation. We screened ~3300 compounds from the ZINC database for C1GALT1 binding. The top 1000 intersections contained 24 drugs. Among them, only 7 were commercially available with minimal side effects to humans and not used in standard anti-cancer therapy. They were montelukast, zafirlukast, telmisartan, cefoperazone, silibinin, piperacillin, and itraconazole. The molecular docking of itraconazole and C1GALT1 was shown (Fig. 4a). We found that only itraconazole prominently increased Tn antigens on surfaces of SAS, OEC-M1, and FaDu cells as revealed by flow cytometry with FITC-VVA (Fig. 4b and Supplementary Fig. 2a). Western blot analysis also confirmed that Tn antigens on cellular proteins were increased in these cells treated with itraconazole (Fig. 4c). Interestingly, we noticed that C1GALT1 protein levels were drastically decreased by itraconazole. By contrast, real-time RT-PCR analysis showed that C1GALT1 messenger RNA expression was not significantly affected (Supplementary Fig. 2b), implying that the effect of itraconazole on C1GALT1 protein levels could be through post-translational modifications. Western blot analysis also showed that this effect was dose-dependent in SAS, OEC-M1, and FaDu cells (Fig. 4d and Supplementary Fig. 2c). Aside from itraconazole, ketoconazole and posaconazole were reported to exhibit anti-cancer activities [23, 24]. Therefore, we examined the effect of other triazole derivatives, including terconazole, ketoconazole, and posaconazole, on Tn expression. Flow cytometry showed that no significant changes in Tn antigens were observed for SAS cells treated with 10 μM of terconazole, ketoconazole, or posaconazole (Supplementary Fig. 2d).

C1GALT1 protein folding occurs in ER and is essential for its stability [15]. Misfolded C1GALT1 is transported to proteasome and then undergoes degradation [25]. To understand the mechanism by which itraconazole promotes C1GALT1 degradation, chloroquine and MG132 were used to inhibit lysosomal and proteasomal degradation pathways, respectively. The results showed that MG132, but not chloroquine, blocked itraconazole-induced C1GALT1 degradation in both SAS and OEC-M1 cells (Fig. 4e). Because the proteasomal degradation pathway is ubiquitination-dependent, we examined whether the ubiquitination of C1GALT1 was increased by itraconazole. We found that itraconazole increased ubiquitinated C1GALT1 compared with DMSO-treated SAS and OEC-M1 cells (Fig. 4f), suggesting that C1GALT1 degradation induced by itraconazole is primarily through the proteasomal degradation pathway.

Next, we examined the interaction of C1GALT1 with itraconazole using cellular thermal shift assays. Results showed that the melting temperature of C1GALT1 was decreased when SAS and OEC-M1 cells were treated with itraconazole (Fig. 4g, left panel). Moreover, under constant melting temperature, C1GALT1 protein levels were decreased by itraconazole in a dose-dependent manner (Fig. 4g, right panel). Taken together, these results suggest that itraconazole directly interacts with C1GALT1 to promote its proteasomal degradation, resulting in decreased C1GALT1 expression in HNSCC cells.

### Itraconazole blocks C1GALT1-mediated malignant phenotypes and EGFR activity

**Fig. 5:**
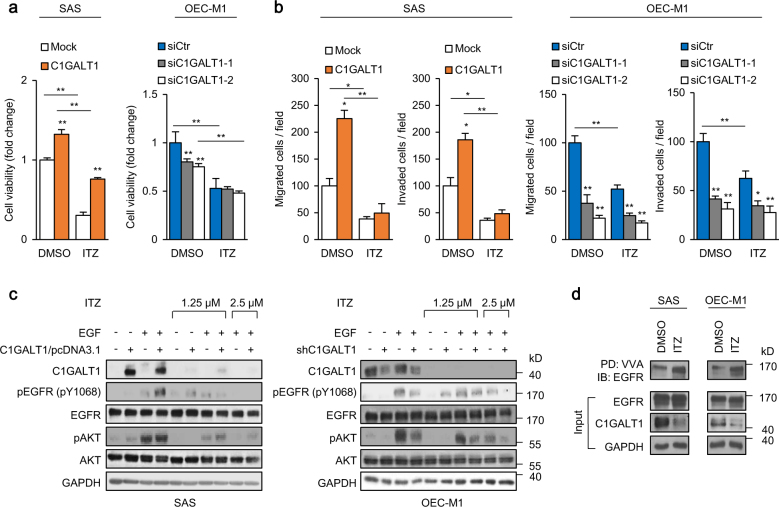
Itraconazole blocks C1GALT1-mediated malignant phenotypes and EGFR activity. **a** Effects of itraconazole on viability of SAS and OEC-M1 cells. SAS cells were transfected with empty pcDNA3.1 (Mock) or *C1GLAT1*/pcDNA3.1A (C1GALT1) and OEC-M1 cells were transfected with non-targeting siRNA (siCtr) or siRNA against *C1GALT1* (siC1GALT1-1, siC1GALT1-2) for 48 h before seeding. Cells were treated with DMSO or 1.25 μM itraconazole (ITZ) after seeding for 24 h (Day 0). Viability was analyzed at day 4 using MTT assay. Data are analyzed by Student’s *t*-test. **P < 0.01. **b** Effects of itraconazole on cell migration and invasion. SAS cells were transfected with empty pcDNA3.1 (Mock) or *C1GLAT1*/pcDNA3.1A (C1GALT1) and OEC-M1 cells were transfected with non-targeting siRNA (siCtr) or siRNA against *C1GALT1* (siC1GALT1-1, siC1GALT1-2) for 48 h before seeding. Cells were treated with DMSO or 1.25 μM itraconazole (ITZ) in the upper chamber of migration and invasion assays. Data are analyzed by Student’s *t*-test. *P < 0.05. **P < 0.01. (**c**) Effects of itraconazole on EGF-induced phosphorylation of EGFR and AKT. SAS cells were transfected with empty pcDNA3.1 (Mock) or *C1GLAT1*/pcDNA3.1A (C1GALT1) and OEC-M1 cells were transfected with non-targeting siRNA (siCtr) or siRNA against *C1GALT1* (siC1GALT1-1, siC1GALT1-2) for 48 h before seeding. Cells were treated with DMSO, 1.25 μM or 2.5 μM itraconazole (ITZ) for 48 h. After starvation for 4 h, the cells were treated with (+) or without (−) EGF (10 ng/mL) for 5 min and collected for western blot analysis. GAPDH was an internal control. **d** Effects of itraconazole on Tn antigen expression on EGFR. SAS and OEC-M1 cells treated DMSO or 2.5 μM itraconazole (ITZ) for 48 h. Five hundred micrograms of proteins from cell lysates were used for each VVA pull-down analysis. EGFR, C1GALT1, and GAPDH from whole cell lysates were shown in the lower panels

We next analyzed whether itraconazole could block C1GALT1-mediated malignant phenotypes in HNSCC cells. MTT, Transwell migration, and Matrigel invasion assays were performed to evaluate the effect of itraconazole on cell viability, migration, and invasion, respectively. The results showed that treatment of itraconazole significantly decreased cell viability, migration, and invasion in SAS and OEC-M1 cells (Fig. 5a, b). Moreover, in SAS cells, the C1GALT1-increased viability, migration, and invasion were significantly reversed by itraconazole. In OEC-M1 cells, itraconazole suppressed these phenotypes in control cells, but to a lesser extent in C1GALT1 knockdown cells.

Because C1GALT1 enhanced EGFR signaling pathways, we examined whether itraconazole could inhibit C1GALT1-increased EGFR activity. The results showed that itraconazole decreased EGF-induced phosphorylation of EGFR and AKT in a dose-dependent manner, which was also tightly associated with C1GALT1 levels in SAS and OEC-M1 cells (Fig. 5c). Moreover, VVA pull-down assays demonstrated that Tn antigens on EGFR were increased by itraconazole (Fig. 5d). These results suggest that itraconazole mediates its suppressive effects on malignant phenotypes and EGFR activity by inhibiting C1GALT1 in HNSCC cells.

### Effects of C1GALT1 and itraconazole on HNSCC tumor growth in vivo

**Fig. 6:**
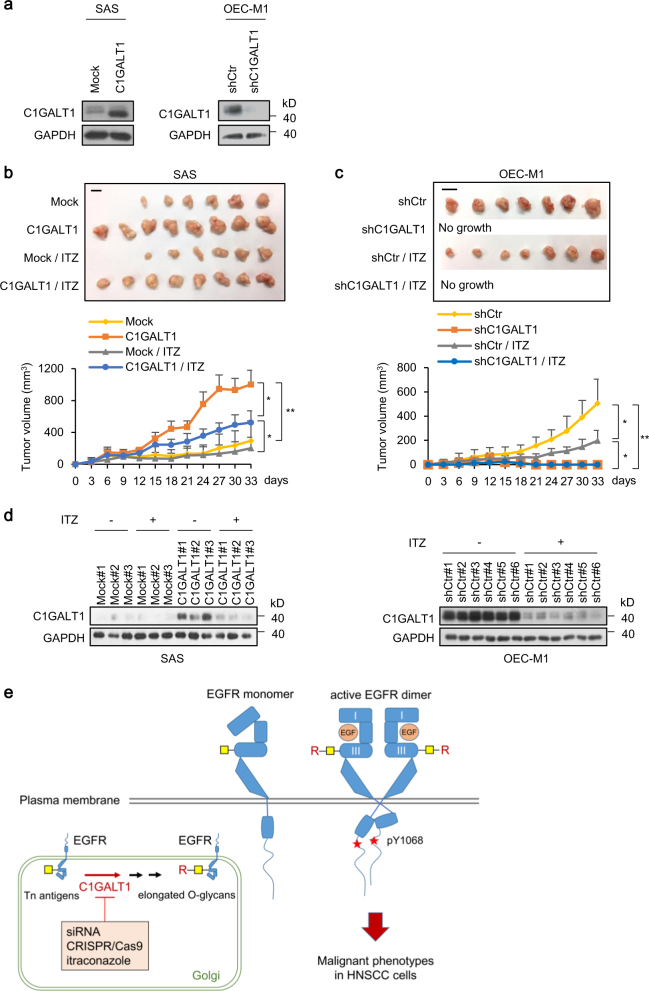
Effects of C1GALT1 and itraconazole on HNSCC tumor growth in vivo. **a** Western blots showing SAS cells stably expressing empty pcDNA3.1 (Mock) or *C1GLAT1*/pcDNA3.1A (C1GALT1) and OEC-M1 cells stably expressing empty pLKO (shCtr) or sh*C1GALT1*/pLKO (shC1GALT1). GAPDH was an internal control. **b** Effects of C1GALT1 overexpression and itraconazole on tumor growth of SAS cells in vivo. Upper panel, images of tumor xenografts. Scale bar, 1 cm. Lower panel, tumor growth curves. Mock or C1GALT1 expressing SAS cells (5 × 10^6^) were injected subcutaneously into NOD-SCID mice treated with control solvent or oral itraconazole (ITZ, 100 mg/kg, twice daily) since day 1 (*n* = 8 for each group). Results are represented as mean ± SD. Data are analyzed by Student’s *t*-test. *P < 0.05. **P < 0.01. **c** Effects of C1GALT1 knockdown and itraconazole on tumor growth of OEC-M1 cells in vivo. Upper panel, images of tumor xenografts. Scale bar, 1 cm. Lower panel, tumor growth curves. shCtr or shC1GALT1 expressing OEC-M1 cells (5 × 10^6^) were injected subcutaneously into NOD-SCID mice treated with control solvent or oral itraconazole (ITZ, 100 mg/kg, twice daily) since day 1 (*n* = 7 for each group). Results are represented as mean ± SD. Data are analyzed by Student’s *t*-test. *P < 0.05. **P < 0.01. **d** Effects of itraconazole on C1GALT1 expression in SAS and OEC-M1 xenografts. Xenografts were collected and C1GALT1 expression was analyzed by western blotting. GAPDH was an internal control. **e** Schematic diagram showing that C1GALT1 modifies O-glycans on EGFR in the Golgi and increases EGF-EGFR binding affinity on the cell surface to promote malignant cell behaviors. Targeting of C1GALT1 using siRNA, CRISPR/Cas9, or itraconazole suppresses C1GALT1-mediated elongation of O-glycans. Note: there are multiple O-glycosites on EGFR, which are simplified in this diagram. Red stars phospho-tyrosine 1068 (pY1068), I domain I, III domain III, yellow squares Tn antigens, R elongated O-glycans, ITZ itraconazole

To evaluate the effect of C1GALT1 and itraconazole on tumor growth, we performed a mouse xenograft model. We first established stable lines of C1GALT1 overexpressing SAS cells and C1GALT1 knockdown OEC-M1 cells (Fig. 6a). The cells were subcutaneously injected into NOD-SCID mice treated with solvent control or itraconazole. The results showed that tumor growth was significantly increased in C1GALT1 overexpressing SAS cells and significantly decreased in C1GALT1 knockdown OEC-M1 cells compared with their controls (Fig. 6b, c). These results suggest that C1GALT1 promotes tumor growth in vivo and silencing C1GALT1 with siRNA is a potential therapeutic strategy for HNSCC.

Next, we assessed whether C1GALT1 inhibitor itraconazole could also suppress tumor growth. In SAS cells, C1GALT1-mediated tumor growth was partially reversed by itraconazole (Fig. 6b), which is closely associated with partially decreased C1GALT1 protein levels in tumors (Fig. 6d, left panel). In OEC-M1 cells, we could not assess the effect of itraconazole in C1GALT1-knockdown cells because of no tumor growth (Fig. 6c). Nonetheless, we observed that itraconazole significantly inhibited tumor growth and C1GALT1 expression in control group (Fig. 6c, d, right panel). These results suggest that inhibition of C1GALT1 with a small molecule compound itraconazole is able to suppress HNSCC tumor growth.

## Discussion

In this study, we explore the role of O-glycosyltransferase C1GALT1 in HNSCC tumors and its potential as a therapeutic target. C1GALT1 expression in HNSCC tumors is positively associated with adverse clinicopathologic factors and is an independent predictor for poor survivals. In HNSCC cells, C1GALT1 promotes malignant phenotypes, including cell viability, migration, invasion, tumor growth, and metastasis. Mechanistically, C1GALT1 modifies O-glycans on EGFR and increases EGF-EGFR binding affinity to promote malignant cell behaviors (Fig. 6e). We also identify a small molecule compound itraconazole as a C1GALT1 inhibitor, which promotes proteasomal degradation of C1GALT1 and phenocopies the effects of C1GALT1 knockdown. In mouse models, we demonstrate that targeting C1GALT1 with genetic or small molecule approach significantly inhibits HNSCC tumor growth.

Glycosylation is one of the most important post-translational modifications of proteins and regulates numerous cellular behaviors [5]. However, the non-template nature of carbohydrate synthesis and diversified oligosaccharides repertoire have impeded investigation of structure and function of site-specific glycosylation. Owing to the SimpleCell technology developed by Dr. Henrik Clausen’s group [26], significant discoveries of novel O-glycoproteins and their O-glycosites have been made. In this technology, COSMC, the C1GALT1-specific chaperone, was knocked out, rendering simplified O-glycans (Tn and sialyl-Tn antigens) on glycoproteins, which were readily identified by ETD-based mass spectrometric analysis. Here we identify one O-glycosite with single HexNAc (GalNAc or GlcNAc) on EGFR from wild-type SAS cells. GalNAc and GlcNAc are stereoisomeric monosaccharides and distinguishing them is challenging for MS. It is highly likely that the HexNAc is GalNAc (Tn antigen) because EGFR from wild-type SAS cells can be pulled down by VVA. Using the SimpleCell technology, we identify additional four O-glycosites with single HexNAc on EGFR from C1GALT1 knockout SAS cells, indicating that EGFR contains at least four GalNAc-type O-glycosites that are modified by C1GALT1. This is the first study to identify GalNAc-type O-glycans on EGFR using MS.

The role of O-linked glycans in ligand-binding affinity was first reported in a G protein-coupled receptor by Bannert et al. [27], who found that O-glycans create an array of negative charges on the surface of C-C chemokine receptor type 5 (CCR5) and allow high-affinity interactions of CCR5 with its ligands. The extracellular domain of EGFR is composed of four subdomains, namely domain I, II, III, and IV. Among which, domains I and III are responsible for ligand binding [28]. Interestingly, our MS results show that 4 out of 5 peptides carrying O-glycans are within domain III (amino acid 310–514) [29]. It is therefore reasonable to speculate that O-glycans within the domain III of EGFR regulate its affinity toward EGF. It was reported that C1GALT1 knockdown decreases galectin-4-mediated but not ligand-mediated EGFR phosphorylation and downregulates EGFR protein levels in prostate cancer cells [19]. By contrast, we show that, in HNSCC cells, C1GALT1 knockdown or knockout decreases EGF-mediated phosphorylation of EGFR without affecting EGFR protein levels. Cells and tissues express distinct repertoires of GALNTs that can regulate O-glycosites and functions of proteins [30–32]. Recently, it was demonstrated that O-glycosylation occurs in a cell-specific manner for hemostatic proteins [32]. The reason that C1GALT1 exhibits different effects on EGFR in prostate cancer and HNSCC cells could be due to cell-specific differential O-glycosites on EGFR. Therefore, deciphering the role of site-specific O-glycosylation in EGFR is critical for understanding the impact of O-glycosylation on RTKs and is underway in our laboratory.

Itraconazole, a common antifungal drug, has been found to exhibit an anti-cancer effect by Kim et al. [33]. Clinical trials of itraconazole as a single agent or in combination therapy suggest benefits in patients with ovarian cancer, recurrent non-small cell lung cancer (NSCLC), prostate cancer, and basal cell carcinoma [34–37]. The known mechanism of its anti-cancer activity is through inhibition of hedgehog, VEGFR2, Wnt, and mTOR pathways [33, 38, 39]. Aftab et al. [38] emphasized its antiangiogenic effect by showing that itraconazole inhibits proliferation of HUVEC endothelial cells, but not NSCLC cells. Here, we propose a new and important role of itraconazole as a C1GALT1 inhibitor. Our results show that itraconazole treatment exhibits similar effects of C1GALT1 knockdown in HNSCC cells. Moreover, in C1GALT1-knockdown cells, itraconazole does not further inhibit cell viability, invasiveness, and EGFR signaling pathways, indicating that itraconazole treatment phenocopies C1GALT1 knockdown. Our results suggest that, although other pathways exist, the anti-cancer effect of itraconazole in HNSCC is mainly through C1GALT1 inhibition. In this study, we prove that targeting C1GALT1 is an attractive strategy for treatment of HNSCC. We would also like to emphasize the druggable property of C1GALT1 by showing that a small molecule compound itraconazole binds to and promotes degradation of C1GALT1. Although C1GALT1 expression is only partially suppressed by itraconazole in mouse models, our results open a new avenue to developing small molecules targeting C1GALT1 with higher specificity and affinity for cancer treatment.

In conclusion, we show that C1GALT1 promotes the malignant behavior of HNSCC and is an independent prognostic factor for poor survivals. In addition, our in vitro and in vivo data support that targeting C1GALT1 is a promising strategy to treat HNSCC.

## Materials and methods

### Tissue samples

Human HNSCC tissues were obtained from National Taiwan University Hospital, Taipei, Taiwan. The use of human tissues was approved by an Institutional Review Board (IRB) at the National Taiwan University Hospital. Informed written consents were obtained from all patients. IRB numbers are 201304078RIND and 201307074RIND.

### Immunohistochemistry

Paraffin embedded tissues were sectioned, deparaffinized, and rehydrated. The sections were incubated with anti-human C1GALT1 monoclonal antibody (1:400, Santa Cruz Biotechnology) at 4 °C for overnight. The UltraVision™ Quanto Detection System (Thermo Fisher Scientific) was used and signals were visualized with DAB Quanto Chromogen provided in the same kit. All sections were counterstained with hematoxylin. The intensity of signals was scored from 0 to 3.

### Plasmid construction

The human full-length *C1GALT1*/pcDNA3.1 was designed as previously described [16]. sh*C1GALT1*/pLKO was obtained from National RNAi Core Facility (Academia Sinica, Taipei, Taiwan, TRCN0000035411). Small guide (sg) RNA for targeting *C1GALT1* in CRISPR/Cas9 system was designed according to database prediction (http://crispr.mit.edu/). The target sequence of sg*C1GALT1* is 5′-GCAACACTTTGTTACAACGC-3′. All constructs described were verified by DNA sequencing.

### Cell cultures and transfection

SAS and OEC-M1 cells were gifts from Dr. Jean-San Chia (College of Medicine, National Taiwan University). FaDu cells were purchased from American Type Culture Collection (ATCC). All cell lines were authenticated using short tandem repeat (STR) profiling analysis in the year 2016. Cells were transiently transfected with 2.5 μg of plasmid with Lipofetamine 3000 (Invitrogen) according to the manufacturer’s protocol. To obtain stable clones, cells were selected with 400 μg/mL G418 or 1 μg/mL puromycin for 14 days. For C1GALT1 knockdown, cells were transfected with 20 nM siRNAs against *C1GALT1* (siC1GALT1-1: 5′-UUAGUAUACGUUCAGGUAAGGUAGG-3′, siC1GALT1-2: 5′-UUAUGUUGGCUAGAAUCUGCAUUGA-3′) using Lipofectamine RNAiMAX (Invitrogen). Non-targeting siRNA (siCtr, 5′-CAACCUCAGCCAUGUCGACUGGUUU-3′, 5′-AAACCAGUCGACAUGGCUGAGGUUG-3′) was used as control. For C1GALT1 knockout, cells were transfected with sg*C1GALT1*/pALL plasmid. C1GALT1-knockout clones were validated by western blotting and DNA sequencing.

### Transwell migration assay and Matrigel invasion assay

Transwell migration assay and Matrigel invasion assay were performed using migration chambers (Corning) and the BioCoat™ Matrigel™ Invasion Chamber system (BD Biosciences), respectively. Cells (2 × 10^4^) in serum-free DMEM were added to an upper chamber. In the lower chamber, 10% fetal bovine serum was used as a chemoattractant. The cells were allowed to migrate or invade for 24 h. The cells on the lower surface of the membrane were fixed with methanol and then stained with 5% crystal violet (Sigma-Aldrich). The number of migrated or invaded cells per field was counted and the mean ± SD was calculated from the number of six random fields.

### Western blot analysis, lectin pull-down assay, and co-immunoprecipitation

Tissue or cell lysates were extracted by homogenizing tissues or cells in NP40 lysis buffer. Proteins were separated on an 8% SDS-PAGE and transferred onto a PVDF membrane. The membranes were blocked in TBST containing 5% bovine serum albumin (Bio-Rad) for 1 h at room temperature and then incubated with primary antibodies against C1GALT1, GAPDH (Santa Cruz Biotechnology), EGFR, pEGFR, pAKT, HA-tag, ubiquitin (Cell Signaling Technology), or AKT (GeneTex Inc.) at 4 °C for overnight. After incubation with a horseradish peroxidase (HRP)-conjugated secondary antibody, protein bands were detected with ECL reagents (GE Healthcare Life Sciences).

For lectin pull-down assay, 500 μg of total proteins from cell lysates with or without 0.4 U/mL neuraminidase (Sigma-Aldrich) treatment were incubated with VVA-conjugated beads (Vector Laboratories) for 18 h at 4 °C with constant rotation. The beads were washed five times with PBS. After boiling beads at 95 °C for 10 min, the pulled-down proteins were separated on an SDS-PAGE.

For co-immunoprecipitation assay, 500 μg of total proteins were incubated with 2 μg of specific antibody for 18 h at 4 °C, followed by adding 50 μL of protein A/G agarose beads (GE Healthcare Life Sciences). After incubation for 3 h, the beads were washed five times with PBS. The beads were resuspended in 30 μl sample buffer and boiled at 95 °C for 10 min. The precipitated proteins were then separated on an SDS-PAGE for western blot analysis.

### Ligand-binding assays

One hundred micrograms of cell lysates were incubated with anti-EGFR antibody (Santa Cruz Biotechnology) coated plates for 18 h. Biotin-EGF (Thermo Fisher Scientific) at different concentrations was added to each well and incubated at 37 °C for 3 h. After incubation with streptavidin-HRP for 1 h, the bound-EGF was detected by o-phenylenediamine dihydrochloride substrates. ELISA reader was used to detect O.D. at 490 nm. The *K*d and Scatchard plot were estimated using binding data.

### In-gel digestion

Gel pieces were washed twice with solutions containing 50% v/v acetonitrile and 50% v/v acetonitrile/25 mM ammonium bicarbonate. The gel fragments were placed in 10 mM dithiothreitol at 60 °C for 1 h for reduction reactions followed by alkylation in 55 mM iodoacetamide in 25 mM ammonium bicarbonate at 65 °C for 1 h at room temperature in the dark. To digest the proteins, 0.1 μg of trypsin (Promega, Madison, WI, USA) was added to each tube, and the samples were incubated at 37 °C for 16 h. After digestion, the supernatant was transferred to an Eppendorf tube. Approximately 20 μL of 50% v/v acetonitrile/5% v/v formic acid was added to each tube to extract the remaining peptides from the gel pieces.

### LC-MS/MS analysis and database search

LC-MS/MS analysis was performed on a Q Exactive Plus mass spectrometer (Thermo Fisher Scientific, Bremen, Germany) equipped with a nanospray interface (Proxeon, Odense, Denmark). Peptides were separated on a nanoAcquity system (Waters, Milford, MA), which was connected to mass spectrometer. Peptide mixtures were loaded onto a 75 μm ID, 25 cm length C18 Acclaim PepMap NanoLC column (Thermo Scientific, San Jose, CA, USA) packed with 2 μm particles with a pore of 100 Å. A segmented gradient in 100 min from 2% to 40% solvent B (acetonitrile with 0.1% formic acid) at a flow rate of 500 nL/min and a column temperature of 35 °C were used. Solvent A was 0.1% formic acid in water. Mass spectrometric analysis was performed in a data-dependent mode with Full-MS (externally calibrated to a mass accuracy of <5 p.p.m., and a resolution of 70,000 at *m/z* = 200) followed by high-energy collision-activated dissociation (HCD)-MS/MS of the top 15 most intense ions. High-energy collision-activated dissociation (HCD)-MS/MS was used to fragment multiply charged ions within a 2 Da isolation window at a normalized collision energy of 27 eV. AGC target at 3*e*6 and 2*e*5 was set for MS and MS/MS analysis, respectively, with previously selected ions dynamically excluded for 90 s. Ions with singly and unrecognized charge state were excluded.

For protein identification, the raw MS/MS data were searched against the Uniprot human database (downloaded on August 2017) using the Mascot and SEQUEST search algorithm via the Proteome Discoverer (PD) package (version 2.2, Thermo Scientific). The search parameters were set as follows: peptide mass tolerance, 10 p.p.m.; MS/MS ion mass tolerance, 0.02 Da; enzyme set as trypsin and allowance of up to two missed cleavages; variable modifications included oxidation on methionine, deamidation on asparagine and glutamine residues, HexNAc on serine and threonine, and carbamidomethylation on cysteine residues. Peptides were filtered based on a 1% FDR.

### Flow cytometry

For detection of Tn antigens on cell surfaces, 1 × 10^6^ cells were incubated with FITC-conjugated VVA (1:200, Vector Laboratories) on ice for 30 min. Immunofluorescence was detected by flow cytometer (Becton-Dickinson).

### Homology modeling and docking simulation

We performed i-tasser (http://zhanglab.ccmb.med.umich.edu/I-TASSER/) and modBase (https://modbase.compbio.ucsf.edu/modbase-cgi/index.cgi) to model the protein structure of C1GALT1. To search for C1GALT1 inhibitors, we performed docking simulation using ZINC database (http://zinc.docking.org/) after C1GALT1 structure formulated.

### Cellular thermal shift assay

Cells (5 × 10^6^) were treated with DMSO or itraconazole for 2.5 h. The cells were washed with ice-cold PBS three times and then suspended in ice-cold PBS containing protease inhibitor cocktail (Sigma-Aldrich). The cells were incubated at room temperature (RT), 53, 55, or 57 °C for 3 min, followed by cooling at RT for 3 min. Cells were snap-frozen in liquid nitrogen and then thawed at 25 °C for three cycles. Cell lysates were then centrifuged at 20,000×*g* for 20 min at 4 °C to remove cellular debris. C1GALT1 in the supernatant was analyzed by western blotting.

### Animals

All animal experiments were approved by Institutional Animal Care and Use Committee of National Taiwan University College of Medicine, Taipei, Taiwan. Female NOD-SCID mice aged 8 weeks were purchased from the National Laboratory Animal Center, Taipei, Taiwan. For tumor growth analysis, 5 × 10^6^ cells were injected subcutaneously at day 0 and tumor sizes were measured every 3 days. For experimental metastasis analysis, 1 × 10^6^ cells were injected into mice through tail veins. Animals were killed at day 60 for evaluation of lung metastasis. To evaluate the effect of itraconazole treatment, solvent control or itraconazole (100 mg/kg, twice daily) was given orally since day 1.

### Statistical analysis

Two-sided Student’s *t*-test was used to analyze the correlation between C1GALT1 expression and clinicopathological characteristics. Kaplan–Meier and Cox regression analysis were performed for survival analysis. *P* < 0.05 is considered statistically significant.

## Electronic supplementary material

Supplementary Figures 1-2

Supplementary Tables 1-3

Supplementary M&M and figure legends
