# Supplementary figures and images for: C1GALT1 predicts poor prognosis and is a potential therapeutic target in head and neck cancer
Source: Oncogene. 2018 Jun 21;37(43):5780–93. doi: 10.1038/s41388-018-0375-0 (PMC6202324; doi:10.1038/s41388-018-0375-0)

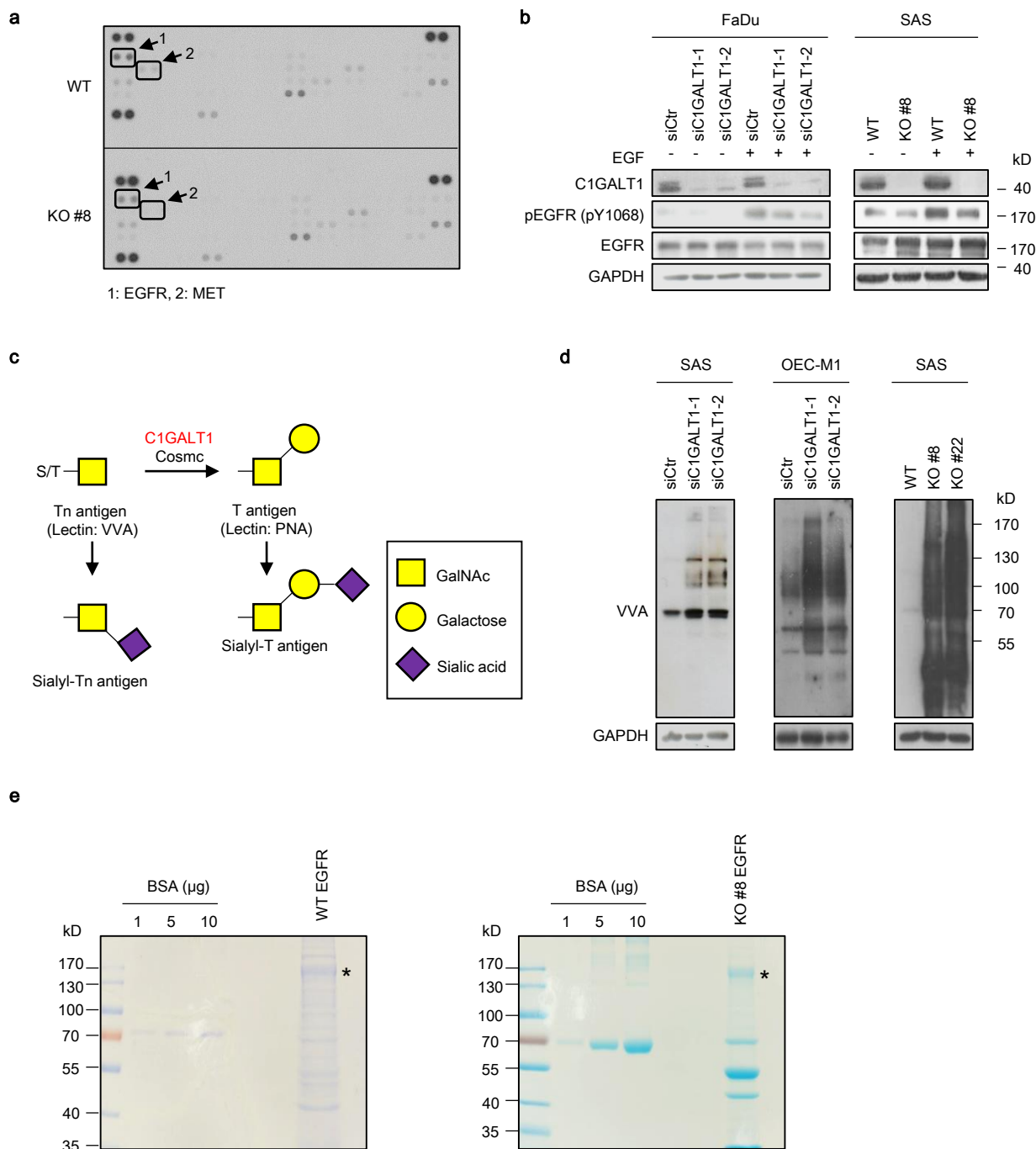

Supplementary Fig.1

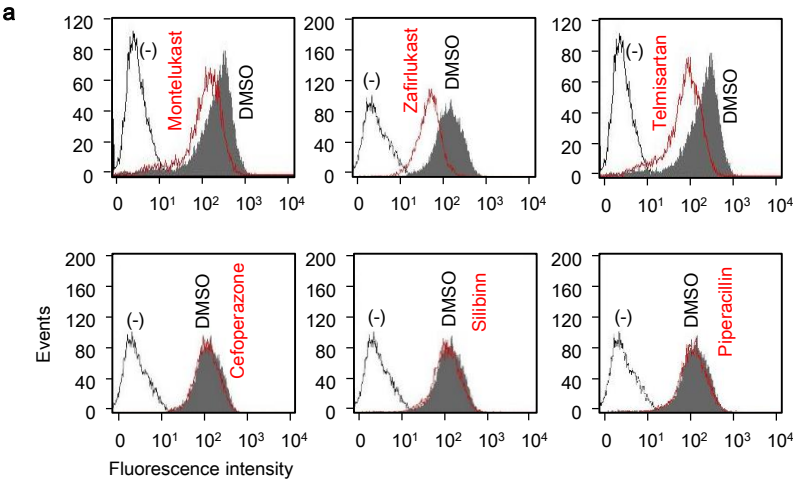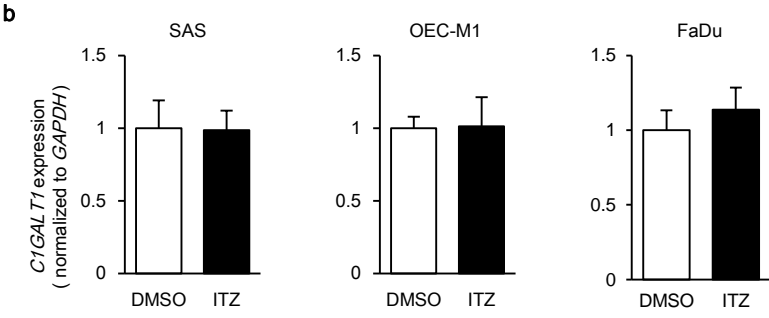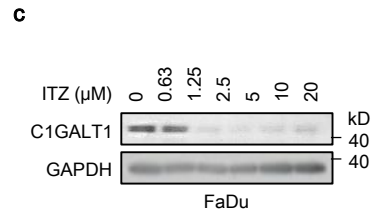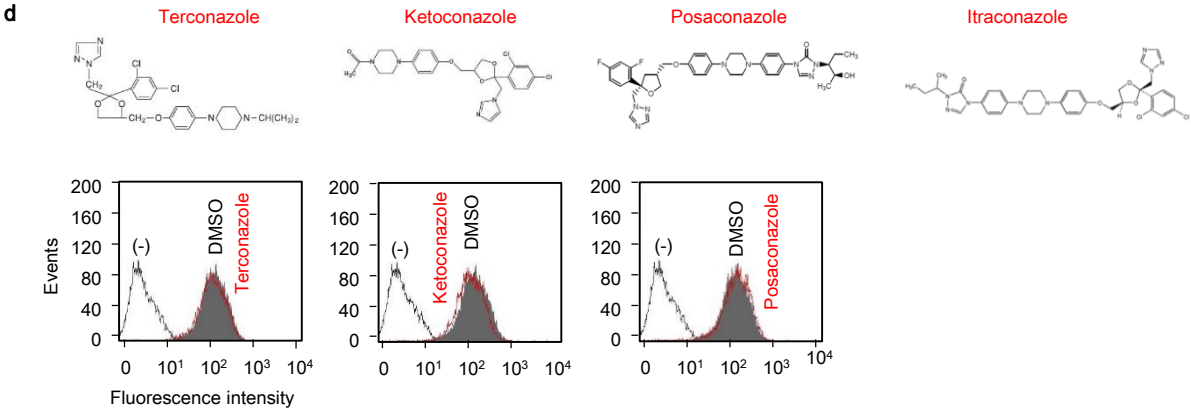

Supplementary Fig.2

Supplement: Supplementary file 1 — Supplementary Figures 1-2 [file 41388_2018_375_MOESM1_ESM.pdf]
