# Supplementary material for: C1GALT1 predicts poor prognosis and is a potential therapeutic target in head and neck cancer
Source: Oncogene. 2018 Jun 21;37(43):5780–93. doi: 10.1038/s41388-018-0375-0 (PMC6202324; doi:10.1038/s41388-018-0375-0)
Supplement: Supplementary file 2 — Supplementary Tables 1-3 [file 41388_2018_375_MOESM2_ESM.docx]

**Supplementary Table 1. Cox-regression analysis of overall survival**

|  | Univariate | | Multivariate | |
| --- | --- | --- | --- | --- |
|  | H | *p* value | H | *p* value |
| Age | 0.79 (0.43-1.47) | 0.46 | NT | |
| T | 1.92 (1.05-3.53) | **0.04** | 1.09 (0.56-2.10) | 0.8 |
| N | 3.45 (1.91-6.24) | **< 0.0001** | 1.79 (0.92-3.51) | 0.09 |
| Grade | 1.29 (0.70-2.36) | 0.42 | NT | |
| LVI | 3.23 (1.78-5.85) | **< 0.001** | 2.53 (1.49-4.29) | 0.3 |
| PNI | 2.93 (1.60-5.39) | **< 0.001** | 2.15 (1.09-4.23) | **0.03** |
| C1GALT1 | 3.76 (1.80-7.82) | **< 0.001** | 2.88 (1.34-6.19) | **< 0.01** |
| score |  |  |  |  |

LVI: lymphovascular invasion; PNI: perineural invasion; NT: not tested; Bold *p* values indicate statistical significance (*p* < 0.05).

**Supplementary Table 2. Cox-regression analysis of disease-free survival**

|  | Univariate | | Multivariate | |
| --- | --- | --- | --- | --- |
|  | H | *p* value | H | *p* value |
| Age | 0.93 (0.56-1.69) | 0.93 | NT | |
| T | 1.86 (1.10-3.15) | **0.02** | 1.20 (0.66-2.18) | 0.54 |
| N | 2.59 (1.54-4.39) | **< 0.001** | 1.33 (0.72-2.43) | 0.36 |
| Grade | 1.43 (0.84-2.44) | 0.19 | NT | |
| LVI | 2.53 (1.49-4.29) | **< 0.001** | 2.53 (1.49-4.29) | 0.42 |
| PNI | 2.98 (1.75-5.05) | **< 0.0001** | 2.30 (1.23-4.31) | **< 0.01** |
| C1GALT1  score | 3.21 (1.73-5.98) | **< 0.0001** | 2.70 (1.40-5.21) | **< 0.01** |

LVI: lymphovascular invasion; PNI: perineural invasion; NT: not tested; Bold *p* values indicate statistical significance (*p* < 0.05).

**Supplementary Table 3. O-glycosylated peptides of EGFR from SAS cells identified by mass spectrometry**

| **Cell type** | **Sequence** | **a.a.** | **Modification** |
| --- | --- | --- | --- |
| Wilde type SAS cells | *DSLSINATNIK* | 347-357 | 1 × HexNAc |
| C1GALT1 knockout SAS cells | *DSLSINATNIK* | 347-357 | 1 × HexNAc |
|  | NCTSISGDLHILPVAFR | 361-377 | 1 × HexNAc |
|  | GDSFTHTPPLDPQELDILK | 378-396 | 1 × HexNAc |
|  | QHGQFSLAVVSLNITSLGLR | 432-451 | 1 × HexNAc |
|  | YADAGHVCHLCHPNCTYGCTGPGLEGCPTNGPK | 609-640 | 1 × HexNAc |

Italic fonts indicate the identical O-glycopeptide of EGFR in both wild type and C1GALT1 knockout SAS cells.
