# Supplementary material for: C1GALT1 predicts poor prognosis and is a potential therapeutic target in head and neck cancer
Source: Oncogene. 2018 Jun 21;37(43):5780–93. doi: 10.1038/s41388-018-0375-0 (PMC6202324; doi:10.1038/s41388-018-0375-0)
Supplement: Supplementary file 3 — Supplementary M&M and figure legends [file 41388_2018_375_MOESM3_ESM.docx]

**Supplementary Materials and Methods**

**RNA extraction and real-time RT-PCR**

Total RNA was extracted with GeneJET RNA purification kit (Thermo Fisher Scientific) according to the manufacturer’s protocol. Two micrograms of total RNA were used in reverse transcription (RT) reaction using the High-Capacity cDNA Reverse Transcription Kit (Applied Biosystems). The cDNA was subjected to real-time PCR. Relative quantity of gene expression was normalized to *GAPDH* and analyzed with MxPro Software (Stratagene). The primer sequences for *C1GALT1* are 5’-TGGGAGAAAAGGTTGACACC-3’ and 5’-CTTGACGTGTTTGGCCTTT-3’. The primer sequences for *GAPDH* are 5’-ACAGTCAGCCGCATCTTCTT-3’ and 5’-GACAAGCTTCCCGTTCTCAG-3’.

**EGFR purification**

Approximately 2 × 10^8^ C1GALT1 knockout cells were harvested. Cell lysates were incubated with cetuximab-conjugated agarose beads at 4℃ for 18 h and then washed with TBST. After wash, immunoprecipitated EGFR was eluted with 0.5 mL of 0.2 M glycine (pH 2.5) and neutralized with 50 μL 1 M Tris (pH 8.8). Eluates were desalted with Amicon (Merck Millipore) and then run on an SDS-PAGE. Separated EGFR was excised and sent for LC-MS/MS analysis (Instrument Center, National Taiwan University, Taipei, Taiwan).

**Supplementary figure legends.**

**Supplementary Fig. 1** C1GALT1 regulates phosphorylation and O-glycosylation of EGFR in HNSCC cells. (**a**) Effects of C1GALT1 on phospho-RTKs. Wild type (WT) or C1GALT1 knockout (KO #8) SAS cells were starved in serum-free DMEM for 12 h and then treated with 10% FBS for 5 min. Cell lysates were harvested for phospho-RTK array assay according to the manufacturer’s protocol. Decreased phospho-EGFR and phospho-MET were indicated by arrows. (**b**) Effects of C1GALT1 on EGF-induced phosphorylation of EGFR. Tyrosine phosphorylation of EGFR (pY1068) was analyzed in C1GALT1 knockdown FaDu cells and C1GALT1 knockout SAS cells (Clone # 8). Cells were starved for 4 h and then treated with (+) or without (-) EGF (10 ng/mL) for 5 min. GAPDH was an internal control. (**c**) Schematic diagram of Tn and T antigen synthesis. S, Serine. T, Threonine. VVA, *Vicia villosa* lectin; PNA, Peanut agglutinin. (**d**) C1GALT1 knockdown or knockout increases Tn antigen expression in HNSCC cells. C1GALT1 knockdown SAS and OEC-M1 cells as well as C1GALT1 knockout SAS cells were harvested. Tn antigen expression was analyzed by Western blot analysis with VVA. GAPDH was the internal control. (**e**) Purification of EGFR from wild type and C1GALT1 knockout SAS cells. Proteins (6 mg) from wild type and C1GALT1 knockout SAS cells were used to purify EGFR using agarose beads conjugated with anti-EGFR antibody. Eluates from C1GALT1 knockout SAS cells were treated with neuraminidase (10 IU) for 30 min to remove sialic acids. Left panel, purified EGFR from wild type SAS cells. Right panel, purified EGFR from C1GAT1 knockout SAS cells. *, EGFR.

**Supplementary Fig. 2** Screening of potential C1GALT1 inhibitors. (**a**) Effects of montelukast, zafirlukast, telmisartan, cefoperazone, silibinin, and piperacillin on Tn antigen expression of the cell surface. Flow cytometry with FITC-VVA on surfaces of SAS cells treated with solvent control DMSO or 10 μM of indicated compounds for 48 h. (-), unstained cells. (**b**) Effects of itraconazole on *C1GALT1* mRNA levels. SAS, OEC-M1, and FaDu cells were treated with solvent control DMSO or 2.5 μM itraconazole (ITZ) for 48 h and *C1GALT1* mRNA levels were analyzed using real-time RT-PCR analysis. (**c**) Effects of itraconazole on C1GALT1 protein levels at various concentrations, as indicated. FaDu cells were treated with DMSO or itraconazole for 48 h and harvested for Western blot analysis. (**d**) Effects of terconazole, ketoconazole, and posaconazole on Tn antigens of the cell surface. Flow cytometry with FITC-VVA on surfaces of SAS cells treated with solvent control DMSO or 10 μM of indicated compounds for 48 h. (-), unstained cells.
